# The pangenome enhances the understanding of the genetic diversity of papaya

**DOI:** 10.1093/hr/uhaf282

**Published:** 2025-10-16

**Authors:** Min Yang, Chenping Zhou, Xiangdong Kong, Ruibin Kuang, Chuanhe Liu, Xiaming Wu, Ze Xu, Han He, Yuerong Wei

**Affiliations:** Institute of Fruit Tree Research, Guangdong Academy of Agricultural Sciences, Key Laboratory of South Subtropical Fruit Biology and Genetic Resource Utilization, Ministry of Agriculture and Rural Affairs, Guangdong Provincial Key Laboratory of Science and Technology Research on Fruit Tree, Guangzhou 510640, China; Institute of Fruit Tree Research, Guangdong Academy of Agricultural Sciences, Key Laboratory of South Subtropical Fruit Biology and Genetic Resource Utilization, Ministry of Agriculture and Rural Affairs, Guangdong Provincial Key Laboratory of Science and Technology Research on Fruit Tree, Guangzhou 510640, China; JiguangGene Biotechnology Co., Ltd, Nanjing 210031, China; Institute of Fruit Tree Research, Guangdong Academy of Agricultural Sciences, Key Laboratory of South Subtropical Fruit Biology and Genetic Resource Utilization, Ministry of Agriculture and Rural Affairs, Guangdong Provincial Key Laboratory of Science and Technology Research on Fruit Tree, Guangzhou 510640, China; Institute of Fruit Tree Research, Guangdong Academy of Agricultural Sciences, Key Laboratory of South Subtropical Fruit Biology and Genetic Resource Utilization, Ministry of Agriculture and Rural Affairs, Guangdong Provincial Key Laboratory of Science and Technology Research on Fruit Tree, Guangzhou 510640, China; Institute of Fruit Tree Research, Guangdong Academy of Agricultural Sciences, Key Laboratory of South Subtropical Fruit Biology and Genetic Resource Utilization, Ministry of Agriculture and Rural Affairs, Guangdong Provincial Key Laboratory of Science and Technology Research on Fruit Tree, Guangzhou 510640, China; Institute of Fruit Tree Research, Guangdong Academy of Agricultural Sciences, Key Laboratory of South Subtropical Fruit Biology and Genetic Resource Utilization, Ministry of Agriculture and Rural Affairs, Guangdong Provincial Key Laboratory of Science and Technology Research on Fruit Tree, Guangzhou 510640, China; Institute of Fruit Tree Research, Guangdong Academy of Agricultural Sciences, Key Laboratory of South Subtropical Fruit Biology and Genetic Resource Utilization, Ministry of Agriculture and Rural Affairs, Guangdong Provincial Key Laboratory of Science and Technology Research on Fruit Tree, Guangzhou 510640, China; Institute of Fruit Tree Research, Guangdong Academy of Agricultural Sciences, Key Laboratory of South Subtropical Fruit Biology and Genetic Resource Utilization, Ministry of Agriculture and Rural Affairs, Guangdong Provincial Key Laboratory of Science and Technology Research on Fruit Tree, Guangzhou 510640, China

## Abstract

Papaya (*Carica papaya* L.) is a nutritionally and medicinally important tropical fruit crop, yet its genetic improvement has been limited by insufficient genomic resources. In this study, we constructed chromosome-level genomes for three key varieties (Zhufeng, T3, and T5) and integrated them with three existing assemblies to build a comprehensive pangenome, including graph-based, linear, and syntelog-based representations. The syntelog-based pangenome revealed 24 453 syntelog groups (SGs). Leveraging resequencing data from 222 accessions aligned to the graph-based pangenome, we identified 26 173 structural variations (SVs), including a functionally relevant 94-bp deletion in the *RETARDED ROOT GROWTH* (*RRG*) gene in the T3 genome. This deletion affects the expression of the *RRG*, resulting in a reduction in its expression level in T3. Further phenotypic analysis showed that *RRG* can influence papaya root length by promoting the proliferation of root meristem cells and inhibiting cell elongation. Additionally, the linear pangenome uncovered 5273 translocations and 1440 inversions, significantly expanding the known SV repertoire in papaya. This study provides a critical genomic resource for deciphering domestication-related traits and accelerating marker-assisted breeding, ultimately advancing the genetic improvement of papaya.

## Introduction

Papaya is one of the most commonly cultivated fruit crops in tropical and subtropical regions around the world [1]. Papaya fruit is juicy and sweet-tasting, and its ripe flesh is rich in vitamins A and C, folate, and calcium. It is also an excellent source of beta-carotene, which can prevent the occurrence of cancer, diabetes, and heart disease [1, 2]. Due to its high nutritional and medicinal value, the global demand for papaya continues to increase [3].

In recent years, remarkable progress has been made in multi-omics research on papaya, encompassing genomics, transcriptomics, metabolomics, and variomics. These studies have provided valuable resources for elucidating its biological characteristics, domestication history, and genetic improvement of papaya. The pioneering work by Ming *et al.* produced the first draft genome of the transgenic ringspot virus-resistant cultivar SunUp, marking a milestone that laid a solid foundation for subsequent studies [4]. Following this achievement, researchers subsequently accomplished high-quality reference genome assemblies for both SunUp and its progenitor Sunset [5]. Genome-wide resequencing-based variomics analyses, including genome-wide association studies (GWAS) and quantitative trait locus (QTL) mapping, have successfully identified numerous SNP and InDel markers significantly associated with important agronomic traits [5–7]. Furthermore, RNA sequencing (RNA-seq) technology has been extensively employed to investigate various biological processes in papaya, including growth and development, abiotic stress responses, and fruit quality formation mechanisms [8, 9]. Although high-quality papaya genomes have been released [5], comparative genomic analyses and trait inheritance studies based on population data for papaya are still limited. In addition, increasing reports have suggested that a single or a few reference genomes are insufficient for representing the full range of genetic diversity of a species [10]. This limits the identification of genetic variants, particularly larger SVs, which play key roles in the genetic determination of agronomical traits [11–15].

To address these challenges, insights from studies of other species provide valuable guidance. In rice, pig, soybean, and other species, pangenome analyses have demonstrated that integrating multiple high-quality genomes yields a more comprehensive representation of within-species variation [16–18]. These efforts have uncovered large-effect structural variations (SVs), presence–absence variations (PAVs), and domestication-related signals that are often undetectable with a single reference genome, thereby facilitating the identification of loci associated with agronomic traits. Building upon these advances, the construction of a papaya pangenome based on diverse cultivated accessions represents a critical step toward elucidating the genetic basis of key traits and enhancing the translational potential of papaya genomic resources.

In this study, based on six high-quality papaya genomes, we constructed a papaya pangenome based on homologues, a graph-based pangenome, and a linear pan-genome. By mapping 222 resequenced papaya accessions to the graph-based pangenome, a high-quality population-scale SV map containing 12 213 SVs was generated, among which 782 potential SVs were identified as being under selection during domestication and breeding. Compared with the graph-based pangenome, the linear pangenome revealed an additional 5273 translocations and 1440 inversions, enhancing our understanding of SV in the papaya genome. In summary, this study analysed genomic variation across different papaya germplasms through genome assembly and comprehensive analysis, identifying SVs associated with functional genes and providing valuable resources for papaya breeding.

## Results

### *De novo* genome assembly and annotation of papaya

**Figure 1:**
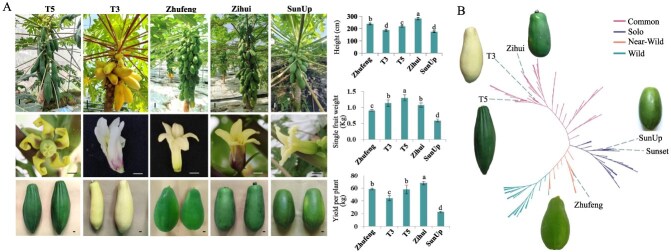
Phenotypic and phylogenetic analysis of papaya. (A) Phenotypic characteristics of T5, T3, Zhufeng, Zihui, and SunUp. Values are means ± SD of three replicates. Statistical significance is indicated by different lowercase letters (*P* < 0.05). Upper left, bar = 10 cm. Middle left and lower left, bar = 1 cm. (B) Papaya phylogenetic tree constructed from SNPs of 222 accessions.

To ensure that the constructed pangenome captures the full range of genetic diversity in papaya, we sequenced the genomes of three papaya varieties (Zhufeng, T3, and T5) that differ in yield, height, fruit weight, colour, and morphology (Fig. 1A; Table S1). These varieties also show significant phenotypic differences compared with the previously reported papaya varieties SunUp and Sunset used in this study. Specifically, the Zhufeng variety is characterized by high yield, oval-shaped fruits, and orange-red flesh when mature. The T3 variety has long oval fruits, with yellow peel in immature fruits and yellow flesh in mature ones. The T5 variety, however, features multi-edged, elongated strip-shaped fruits, and its mature flesh is orange-yellow. The phylogenetic tree shows that these papaya germplasms are distributed on different branches (Fig. 1B). For example, the Solo variety SunUp is a transgenic disease-resistant strain and is a representative of small-fruit papayas; T3 and T5 have larger fruits and diverse shapes, and may carry unique stress resistance or quality-related genes; Zhufeng, as a wild close-source species, serves as a reference for genetic diversity and may contain adaptive genes such as disease resistance and insect resistance. We also completed the genome sequencing and assembly of Zihui, another papaya variety that we previously selected through hybrid breeding and that significantly differ from Zhufeng, T3, T5, and SunUp in key agronomic traits [7], such as fruit morphology and yield (Fig. 1A; Table S1).

Using PacBio HiFi sequencing, we generated 20.72 to 38.92 Gb of data, with sequencing depths ranging from 60.9× to 115.75×. Using these HiFi reads and ultradeep Hi-C data (>150×), we constructed four high-quality papaya genomes, with an average genome size of 337.54 Mb. Approximately 98.5% of the sequences were anchored to nine chromosomes, with 11 to 16 gaps and 14 to 16 telomeres identified per genome. A phylogenetic tree constructed using SNP information from 222 papaya accessions indicated that the varieties we sequenced presented a wide range of genetic relationships. Based on our previous research [7], the four papaya varieties assembled in this study, as well as the previously reported genetically modified cultivar SunUp and its progenitor Sunset varieties [5], were genetically distant on the evolutionary tree, making them more representative (Fig. 1B). We also incorporated the genomes of these two varieties into the comparison and analysis of this study. We used the genome improvement tool RagTag to upgrade the SunUp and Sunset genomes using Zhufeng as a reference, resulting in genome sizes of 350.35 and 351.48 Mb, respectively (Figs S1 and S2). Genome completeness was assessed using BUSCO, with scores ranging from 97.7 to 99.1%, indicating high integrity of the gene regions. Between 42.59% and 54.75% of the sequences in these genomes were predicted to be TEs. To maintain consistency in downstream analyses, we applied the same standardized MAKER2 pipeline to annotate protein-coding genes across all six genomes, resulting in the identification of 19 775 to 20 523 protein-coding genes, with an average CDS length of 1223 bp (Table 1).

**Table 1:** Construction and annotation of papaya genomes

Accession:	Zhufeng	T3	T5	Zihui	Sunset	SunUp
PacBio HiFi data (Gbp)	26.58	38.92	31.95	20.72		
HI-C data (Gbp)	83.82	105.77	128.72	115.41		
Assembly length (Mbp)	337.98	336.25	335.69	340.23	350.35	351.48
Contig N50 (Mbp)	35.45	30.61	30.55	29.14		
Contig N90 (Mbp)	13.29	11.65	11.42	8.25		
Gap number	13	11	14	16	108	221
Telomere	16	14	16	15		
Chromosome anchoring rate (%)	98.59	98.45	99.11	97.86	94.25	93.24
Gene number	19 893	19 775	19 893	19 819	20 523	20 370
TE number	237 668	236 446	203 491	164 534	243 321	375 071
BUSCO	97.7	97.9	97.9	97.9	99.1	98.8

### Syntelog-based papaya pangenome

**Figure 2:**
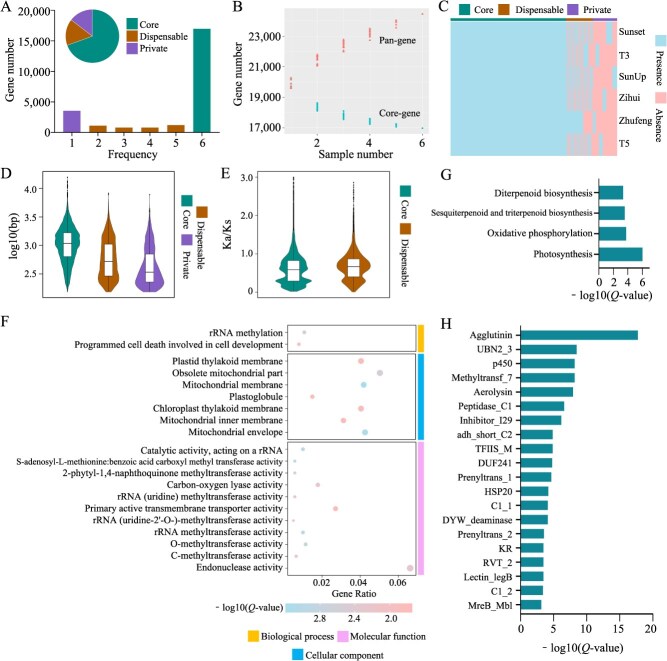
Pangenome and core gene analysis of six papaya accessions. (A) Distribution and proportion of core, dispensable, and private genes in the syntelog-based papaya pangenome. (B) Trends of the pan genes and core genes in the syntelog-based papaya pangenome as the sample size increased. The numbers of pan genes and core genes were calculated by randomly sampling genomes 100 times for each sample on the x-axis. (C) PAV in SGs across the six papaya genomes. (D) Distribution of CDS lengths among core, dispensable, and private SGs. (E) Distribution of Ka/Ks ratios for genes in core and dispensable SGs. (F) GO enrichment analysis of dispensable genes. (G) KEGG enrichment analysis of dispensable genes. (H) Pfam enrichment analysis of dispensable genes.

To more accurately identify highly similar paralogues and orthologues, we constructed a syntelog-based papaya pangenome using the genomes of three newly *de novo* assembled genomes, one previously sequenced and assembled genome and two improved genomes. This generated 120 273 genes from the six genomes, which were grouped into 24 453 SGs (Table S2). Depending on presence/absence variation (PAV), these SGs were classified into core SGs that were present in all six genomes, dispensable SGs present in two to five genomes, and private SGs present in only one genome. Specifically, there were 16 975 core SGs (69.42%), 3916 dispensable SGs (16.01%), and 3652 private SGs (14.57%) (Fig. 2A). As the number of genomes increased, the number of core SGs tended to decrease (Fig. 2B). Across the six papaya genomes, core SGs accounted for 83.85% to 86.54% of the SGs, dispensable SGs for 10.71% to 12.59%, and private SGs for 2.07% to 4.38% (Fig. 2C; Fig. S3). Nucleotide-binding leucine-rich repeat receptors (NLRs) are an important class of resistance genes in plants that play a critical role in plant immune responses against pathogens. Due to their rapid evolution and high diversity, NLRs are a key focus for studying genomic variation and adaptation in plants. In our syntelog-based pangenome, we identified 18 NLR SGs with RGAugury [19], which is 9 more than found in the single Zhufeng genome. This expanded diversity of NLRs highlights the value of constructing a pangenome, which captures a more comprehensive representation of resistance gene diversity that would be missed when analysing a single reference genome. By focusing on NLRs, we aimed to provide insights into the genetic basis of disease resistance in papaya, which is crucial for breeding programs aimed at improving crop resilience (Table S3).

We found that the CDS length of core genes was significantly longer than that of dispensable and private genes (Fig. 2D), suggesting that dispensable and private genes may undergo domain gain-and-loss variation. Domain annotation of protein sequences revealed that 80% of core genes could be annotated with domains, whereas only 52.6% and 31.7% of dispensable and private genes could be annotated, respectively (Table S4). This result indicated that, compared with core genes, a greater proportion of dispensable and private genes experienced domain gain-and-loss variation. This could be related to selective pressures during breeding [20]. To further understand the selective pressures acting on these genes, we calculated the Ka/Ks ratios between genes within the same SG and found that the Ka/Ks ratios among genes in dispensable SGs were significantly greater than those in core SGs (Fig. 2E).

To further explore the functions of core, dispensable and private genes, we conducted enrichment analyses. GO enrichment analysis revealed that dispensable genes were involved primarily in functions such as rRNA methyltransferase activity, carbon–oxygen lyase activity, and 2-phytyl-1, 4-naphthoquinone methyltransferase activity (Fig. 2F). KEGG pathway enrichment analysis indicated that dispensable genes were involved mainly in photosynthesis, oxidative phosphorylation, sesquiterpenoid and triterpenoid biosynthesis, and diterpenoid biosynthesis pathways (Fig. 2G). The functional analysis of dispensable genes suggested that genomic differences between varieties may be related primarily to photosynthesis and certain secondary metabolites. In particular, the sesquiterpenoid and triterpenoid biosynthesis (including genes such as *TPS21*, *SQE3*, and *BAS*) and diterpenoid biosynthesis (including genes such as *GA3*, *CYP88A3*, and *KAO2*) pathways synthesize metabolites with diverse chemical structures that play various biological roles in plants, such as insect resistance and symbiosis with microorganisms [21, 22]. These pathways also have various medicinal properties [23]. Additionally, Pfam annotation enrichment analysis of dispensable genes revealed that their protein functional domains are enriched primarily in gene families such as agglutinin (25 genes) and P450 (58 genes) (Fig. 2H). Among them, the agglutinin gene was largely missing in the T3 variety, and the P450-related genes showed different degrees of PAV variation in the six varieties. The P450 gene family is a crucial group of oxidase genes involved in the synthesis of secondary metabolites such as triterpenoids and diterpenoids [24], which further suggests potential differences in secondary metabolite synthesis pathways among the genomes of different papaya varieties. These findings provide insights into the differences in stress resistance and nutritional value among papaya varieties. Enrichment analysis of core and private genes revealed that core genes were involved mainly in plant resistance pathways (e.g. plant hormone signal transduction and alanine, aspartate, and glutamate metabolism), whereas private genes were enriched primarily in photosynthesis (Zhufeng, T5, Sunset, and SunUp) and protein processing in the endoplasmic reticulum (Zihui) (Tables S5 and S6).

### Graph-based pangenome construction and population-scale SV identification

**Figure 3:**
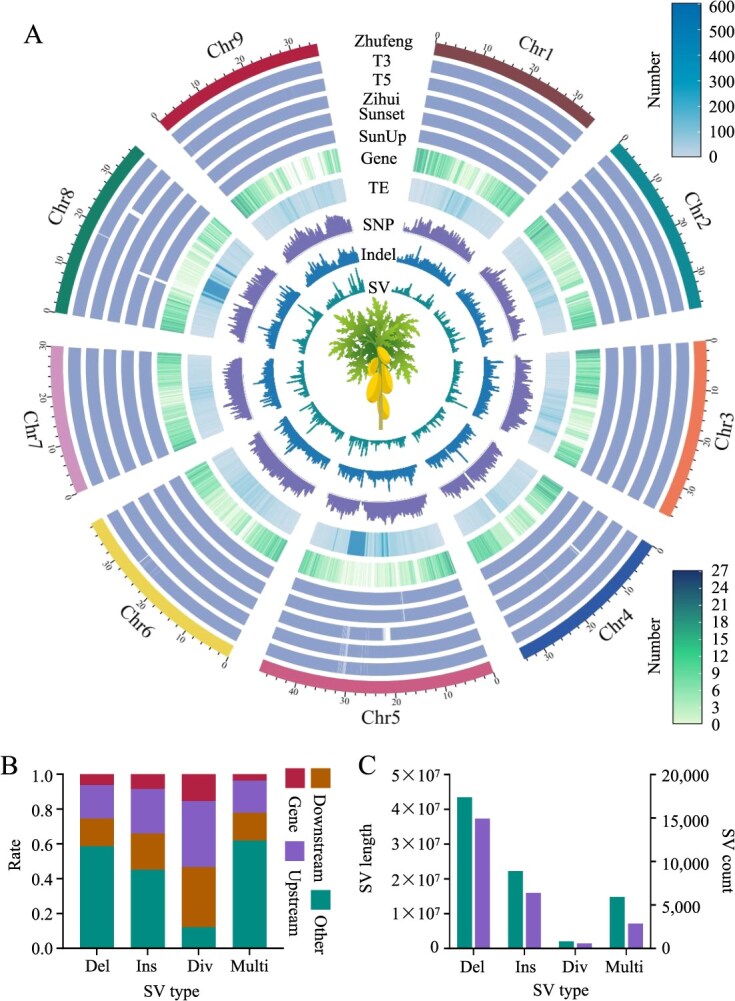
Papaya graph-based pangenome and population-scale SVs. (A) Genome features and variation in the graph-based pangenome. T3, T5, Zihui, Sunset, and SunUp represent the collinearity analysis between the Zhufeng genome and each the corresponding genome, with the colored regions indicating areas of collinearity. ‘Gene’ represents the number of genes in the Zhufeng genome. ‘TE’ indicates the number of TEs in the Zhufeng genome. ‘SNP’, ‘Indel’, and ‘SV’ represent the counts of these variations identified in this study. Windows: 1 Mb. (B) Positions of different SV types relative to genes. ‘Del’, ‘Ins’, ‘Div’, and ‘Multi’ represent deletion, insertion, divergent, and multiallelic, respectively. (C) Number and length distribution of different SV types.

A graph-based pangenome can integrate genomic variation information across multiple individuals within a species. In this study, we constructed a papaya graph-based pangenome by integrating the genomes of five different papaya varieties using the Zhufeng genome as a reference. The resulting graph-based pangenome had a size of 371.56 Mb, comprising 197 069 nodes. Among these nodes, core nodes (shared by all the genomes) accounted for 301.59 Mb (81.17%), whereas variable nodes (present in five or fewer genomes) accounted for 69.97 Mb (18.83%) (Fig. 3A). The graph-based pangenome contained a total of 12 213 bubbles (representing SVs in the genome), with SV lengths ranging from 50 to 360 106 bp. The mean and median lengths of these SVs were 2833 and 276 bp, respectively. Overall, the length of nodes within each genome that corresponded to variable nodes in the graph-based pangenome ranged from 28.28 to 31.64 Mb (Fig. S4). Additionally, these nodes overlapped with 1438 protein-coding genes and 18 445 TE regions, suggesting that changes in TEs are a significant source of the rich SV diversity found in papaya genomes.

**Figure 4:**
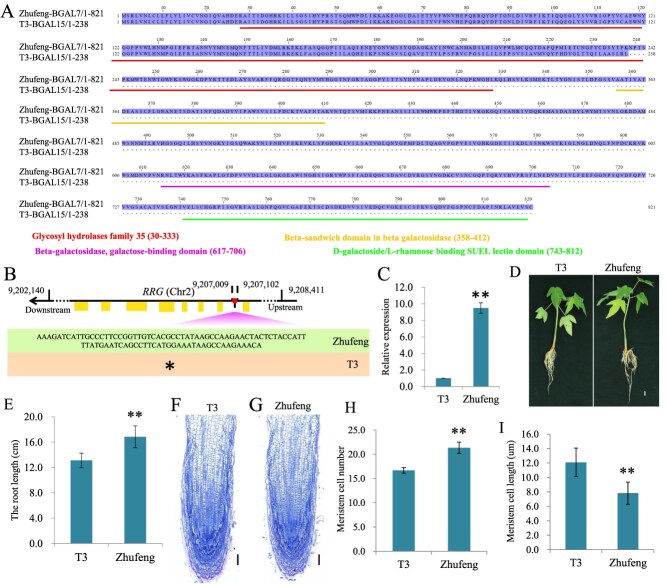
SVs in functional genes identified through pangenome analysis. (A) Alignment of BGAL7 and BGAL15. Domains are represented by lines. (B) In the T3 genome, there was a 94-bp deletion in the first intron of the *RRG* gene on chromosome 2. (C) RT-qPCR results of *RRG* expression levels in T3 and Zhufeng papaya. (D) Root length comparison of T3 and Zhufeng papaya tissue-cultured seedlings at the same growth stage; bar = 1 cm. (E) Statistical analysis of root lengths of T3 and Zhufeng papaya seedlings at the same growth stage. (F-G) Cytological structures of seedling roots in T3 and Zhufeng. Scale bar = 100 μm. (H) The average cell numbers in the root meristem of T3 and Zhufeng. (I) The average cell length in root meristem of T3 and Zhufeng. Mean values ± SDs are shown for three biological replicates (*n* = 3); ^*^P < 0.05, ^**^P < 0.01.

We mapped the 222 resequenced papaya accessions (50 accessions generated from our previous studies and 172 from NCBI; Table S7) to the graph-based pangenome and performed SV genotyping. After filtering out genotypes with a maximum missing value ≤0.3 and a minor allele frequency (MAF) ≥0.01, we identified a total of 26 173 high-confidence SVs (Table S8). This not only surpassed the reliability of the SV calling results obtained using a single papaya genome as a reference but also resulted in a higher SV count, exceeding the previously identified 8083 SVs [6]. The population-scale SVs identified in this study could be categorized into four different types: biallelic (insertions, deletions, and divergent alleles) and multiallelic (Fig. 3B). Among these, insertions and deletions were the most prevalent types of SVs, with 55.52% located in potential regulatory regions of genes (within 2 kb upstream or downstream of the gene) and 8.37% overlapping within protein-coding sequences (Fig. 3C). These SVs alter gene structure and may have potential impacts on gene expression.

### Key functional gene variations revealed by the pangenome

The phenotype of T3 is notably distinct from that of other papaya varieties, particularly in fruits, which exhibit a characteristic yellowing (Fig. 1A; Table S1). Pangenomes assembled from multiple high-quality genomes can directly identify mutations in functional genes. Functional annotation of the syntelog-based papaya pangenome revealed that genes within the same SG consistently mapped to the same KO terms. However, one particular SG (SG0015078) was an exception: the *T3Cp15069* gene within this SG was annotated with a different KO term in the T3 genome than in the other genomes. In the T3 genome, this gene was annotated as *β*-galactosidase 15 (*BGAL15*), whereas in the other five genomes, it was annotated as *β*-galactosidase 7 (*BGAL7*). Previous studies have shown that *BGAL7* and *BGAL15* share a common ancestor [25], so we further cloned the *BGAL15* and *BGAL7* genes and performed amino acid sequence alignment. The results indicated that compared with *BGAL7*, *BGAL15* has multiple functional domains missing (Fig. 4A；Fig. S5), which we speculate might have been lost during the evolutionary process.

Interestingly, through the analysis of SVs in the graph-based pangenome, we discovered a 94-bp deletion in the first intron of the *RETARDED ROOT GROWTH* (*RRG*) gene on chromosome 2 in the T3 genome, and this deletion was further confirmed by sequence amplification and sequencing (Fig. 4B). Although the deletion of an intron does not directly alter the encoded protein, it might influence splicing efficiency or regulatory elements within the intron, potentially affecting gene expression levels or isoform diversity [26, 27]. Therefore, we first cloned the coding sequences (CDS) of *RRG* from Zhufeng, T3, T5, and Zihui papaya, and found that their CDS were identical. Subsequently, we conducted *RRG* gene expression level detection and phenotypic analysis using T3 and Zhufeng as materials. RT-qPCR results showed that the expression level of *RRG* in Zhufeng was 9.5-fold higher than that in T3 papaya (Fig. 4C). Meanwhile, since previous studies in the model plant *A. thaliana* have shown that *RRG* plays an important role in the regulation of root meristem cell division and affected root development [28], we explored whether the expression pattern of *RRG* in papaya is related to root development. Firstly, we compared the root lengths of seedlings of the two varieties at the same growth stage and found that the root length of T3 was significantly shorter than that of Zhufeng (Fig. 4D–E). Furthermore, we examined and analysed the average cell number and cell length in the root meristem of T3 and Zhufeng within the same region (Fig. 4F–I). We found that the average cell numbers in the root meristem of T3 and Zhufeng were 16.6 ± 0.6 and 21.3 ± 1.15, respectively, indicating a reduction in cell proliferation in T3 (Fig. 4H). In contrast, the cell length in root meristem of T3 (12.1 ± 1.9 μm) was significantly increased compared with the Zhufeng (7.8 ± 1.5 μm) (Fig. 4I), suggesting that cell expansion in the meristematic cells was enhanced in the T3. These above results suggested that the function of RRG in papaya may be similar to that in Arabidopsis, influencing root growth by promoting cell proliferation and inhibiting cell expansion in the root meristem.

### SV polymorphisms among papaya populations

**Figure 5:**
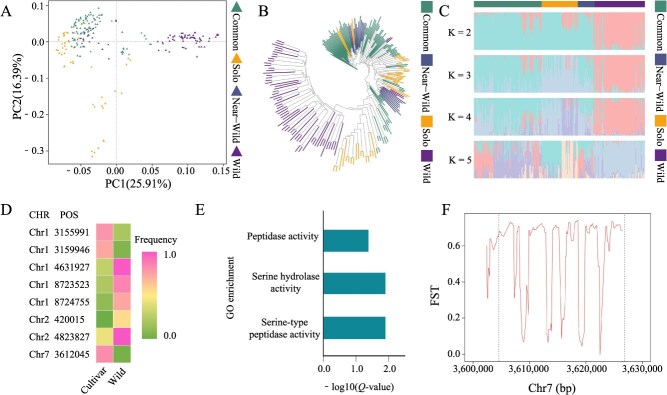
Population analyses based on SVs. (A) PCA of different papaya populations based on SVs. (B) Neighbour-joining tree of different papaya populations based on SVs. (C) Population structure analysis of different papaya populations based on SVs. (D) Frequency of SVs affecting protein-coding genes in cultivated and wild populations. (E) GO enrichment analysis of protein-coding genes overlapping with SVs. (F) SNP-based Fst analysis of the *DEG15* gene and the regions 2 kb upstream and downstream between cultivated and wild papaya varieties. The dotted line indicates the gene region.

A principal component analysis (PCA) based on SVs revealed a clear distinction between cultivated and wild populations (Fig. 5A). A phylogenetic tree constructed using population-scale SVs further illustrated the complex relationships among different accessions (Fig. 5B). Notably, the solo and common populations among the cultivars presented interspersed distributions on the phylogenetic tree. This observation may be attributed to their classification based on fruit size rather than on factors more closely related to phylogenetic relationships, such as geographical distribution or genetic background, indicating that such a classification may not fully capture genetic differences. Population structure analysis also revealed significant disparities between wild and cultivated varieties (Fig. 5C), which contrasts with previous findings based on SNPs. This discrepancy could be due to the greater impact of SVs on genes than of SNPs on genes, leading to stronger selective pressures on SVs and resulting in inconsistent population structures between the two markers. These findings offer new insights into the population structure dynamics of papaya beyond those possible via traditional SNP-based genotyping approaches. The study identified 782 SVs with a frequency difference greater than 0.5 between wild and cultivated populations, among which 114 SVs overlapped with 79 protein-coding genes (Table S9). GO enrichment analysis revealed that six genes, including Serine Protease (*DEG15*), natural resistance-associated macrophage proteins (*NRAMP2*), serine carboxypeptidase-like 27 (*SCPL27*), and serine carboxypeptidase-like 42 (*SCPL42*), were significantly enriched in three GO terms: serine-type peptidase activity, serine hydrolase activity, and peptidase activity (Fig. 5D, E). These genes have been found to be involved in plant insecticide resistance, metal transport, and other functions [29–32]. Notably, *DEG15* gene knockout studies have demonstrated its role in pesticide resistance in *A. thaliana* [29]. Further analysis of the Fst and π values within 2-kb upstream and downstream of *DEG15* with SNPs indicated substantial genetic divergence between cultivated and wild populations (Fig. 5F; Fig. S6). These results suggest that *DEG15* may have undergone selection during the domestication process of papaya, highlighting its potential role in the adaptation and improvement of cultivated varieties.

**Figure 6:**
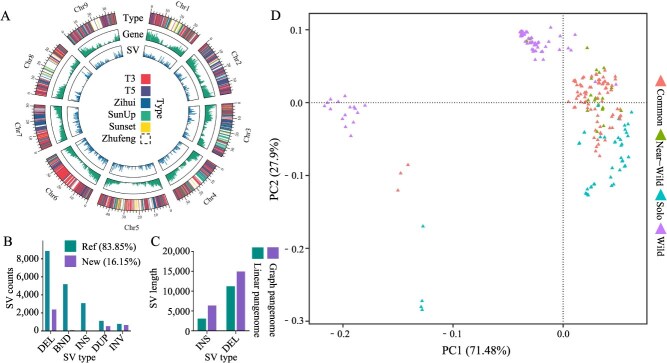
SVs identified in the linear and graph-based pangenomes. (A) Linear pangenome using Zhufeng as the reference genome. From the outer to inner circles, the figure displays the sources of SVs, the distribution of pangenome genes across chromosomes, and the distribution of SVs along chromosomes. Windows: 1 Mb. (B) Number of different types of SVs. ‘DEL’, ‘BND’, ‘INS’, ‘DUP’, and ‘INV’ represent deletions, translocations, insertions, duplications, and inversions, respectively. (C) Number of insertions and deletions identified using the linear and graph-based pangenomes. ‘Linear pangenome’ and ‘graph-based pangenome’ represent SVs identified based on the linear and graph-based pangenomes, respectively. ‘INS’ and ‘DEL’ represent insertion and deletion SVs, respectively. (D) PCA based on SVs identified in the linear pangenome.

### Comparison of SVs identified using the linear and graph-based pangenomes

Graph-based pangenomes allow for the integration of genomic variations from different individuals into a single reference framework, providing a more comprehensive capture of complex SVs. However, due to the complexity of their format, researchers face challenges in analysing and using graph-based pangenomes. Additionally, because SV calling in graph-based pangenomes relies on alignment against graph paths, these pangenomes tend to perform poorly in detecting complex SVs, such as translocations and inversions [33, 34]. Therefore, we compared the SV calling results using both the graph-based pangenome and the linear pangenome as references. First, we constructed a linear pangenome using the Zhufeng genome as a reference. We found 2800 new sequences from the genomes of five other papaya varieties integrated into the Zhufeng-based linear pangenome (Fig. 6A; Table S10), including 13 new genes (Table S11). The average, median, maximum, and total lengths of these new sequences were 2010 bp, 301 bp, 130 002 bp, and 5.63 Mbp, respectively. In the linear pangenome, we analysed the relative positions of these new sequences inserted into the Zhufeng genome and found that 38.75% of the new sequences overlapped with the regions 2 kb upstream and downstream of genes, whereas 13.57% overlapped with gene regions. These findings suggested that the linear pangenome also captured a significant number of newly added functional sequences in the genome, providing insights for future functional gene studies.

Compared with the graph-based pangenome, the linear pangenome detected an additional 5273 and 1440 translocation and inversion SVs, respectively (Fig. 6B). We mapped the resequencing data from 222 papaya accessions to the linear pangenome for SV calling. Using thresholds of a maximum missing value ≤0.3 and MAF ≥0.01, we identified 22 703 SVs (including insertions, deletions, duplications, translocations, and inversions) in the population using the linear pangenome as a reference. Although the graph-based pangenome could detect more SVs overall, particularly complex SVs such as translocations and inversions, the linear pangenome provided a more complete view of these variations (Fig. 6C). Similar to the population-scale SV analysis based on the graph-based pangenome, the PCA of SVs based on the linear pangenome also revealed clear distinctions between cultivated and wild populations (Fig. 6D). Notably, among the 6713 detected translocation and inversion SVs, 208 exhibited frequency differences between wild and cultivated populations. These SVs were associated with genes such as *AE7*, *SYN2,* and *rps11* [35–37]. This complemented the limitations of the graph-based pangenome approach, providing a more comprehensive understanding of the differences between various papaya populations. Moreover, the Fst analysis of SVs identified using different methods between cultivated and wild populations indicated that the linear pangenome better highlighted the divergences between these populations (Fig. S7).

## Discussion

With the advancement of third-generation sequencing technologies, large-scale identification of SVs has been successfully used to identify valuable genes in multiple plant species [10, 38, 39]. In recent years, progress has been made in genomic research on papaya, with a primary focus on sex chromosomes, the impact of transgenics on the genome, and the domestication history of papaya through population genomics [5]. However, the significant differences between individuals within a species, which can be revealed through comparative genomics, should not be overlooked [40]. In this study, we selected six representative papaya varieties with distinct phenotypes (Zhufeng, T3, T5, Zihui, Sunset, and SunUp), constructed a graph-based pangenome, and further identified 12 213 SVs. Due to the difficulty in obtaining materials, this study did not include a wild papaya chromosome-level genome, limiting the identification of more SVs. However, the use of the graph-based pangenome as a reference enabled precise SV identification at the population level using second-generation sequencing data [41]. We collected whole-genome resequencing data from 222 accessions, including wild papaya, and generated for the first time high-precision SV maps in papaya to identify selection signals. These SVs have greater utility than previously published population-scale SVs in papaya [6]. The 782 SVs that showed frequency differences (>0.5) between wild and cultivated populations provide a rich resource of population-scale SVs. By comparing these with the genomic coordinates of protein-coding genes and analysing the graph-based pangenome, this research offers valuable insights for gene discovery among numerous varieties with phenotypic differences.

The construction of multiple high-quality papaya genomes has provided new possibilities for in-depth exploration of genomic resources across different varieties. Typically, for multiple high-quality genomes, downstream analyses can be conducted in two ways: by constructing a gene-based pangenome to capture the complete gene set of a species or by building a graph-based pangenome to capture SVs. In recent studies, gene-based pangenome construction has often involved iterative analysis of homologous genes using MCScan or identification of homologous gene clusters using Markov clustering [42]. In contrast, this study employed a syntelog-based pangenome construction using the genomic coordinates of gene models, providing a more accurate method for identifying homologous gene clusters [43]. Although the study lacked sufficient genomes, such as those from wild papaya, to perform a more comprehensive analysis, we still identified 29 dispensable genes related to the synthesis of important secondary metabolites. These findings suggest that these genes exhibit plasticity within the genomes of different papaya varieties, possibly due to selective pressures during breeding. Furthermore, through the identification of variety-specific genes, multiple candidate genes were identified, such as the *RRG* and *BGAL7* in T3. Notably, the structural variations (SVs) in the *RRG* of T3 (94-bp deletion) may underlie its lower *RRG* expression level compared to other varieties, and this expression difference affects the proliferation of root meristem cells and cell elongation, thereby influencing the root length of papaya. These findings lay the foundation for developing variety-specific genetic resources.

Using a graph-based pangenome as a reference for precise SV calling in populations can significantly enhance the genetic analysis of quantitative traits. For example, conducting SV-based GWASs by incorporating phenotypic data can help identify missing heritability that might be overlooked in SNP-based GWASs [38]. However, due to limited resequencing data and associated phenotypic information available in this study, we lacked the statistical power to identify SVs significantly associated with phenotypes. This highlights the need for further collection of samples and phenotypic data. Nonetheless, the use of SVs as genotypes for comparing wild and cultivated populations offers a valuable opportunity to explore the potential selective pressures on SVs during the domestication and breeding of papaya. We identified 114 SVs that overlapped with 79 protein-coding genes, providing direct references for selecting specific genes for molecular biology research. In particular, with new advancements in sample collection and phenotypic measurements, SVs that show frequency differences between wild and cultivated populations could be further integrated with SV-GWASs to determine their impact on specific phenotypes.

Although graph-based pangenome construction has become a common method for pangenome analysis based on high-quality genomes, its complex format often hinders in-depth understanding and application of the data. Recent studies have proposed a linear pangenome approach to integrate multiple high-quality genomes. While graph-based pangenomes can detect insertions, deletions, and multiallelic variants, they are limited in identifying certain SVs. Using the linear pangenome, we identified 8370 new SVs, including duplications, translocations, and inversions, which could not be captured by the graph-based approach. Furthermore, an inversion associated with the *AE7* gene differed in frequency between wild and cultivated populations. These findings not only enrich the SV map of papaya but also provide a valuable opportunity for exploring the value of combining various pangenome analysis methods in population studies.

## Materials and methods

### Plant materials and sequencing

The Zhufeng and Zihui papaya varieties were developed by the Guangdong Academy of Agricultural Sciences, China [44]. We collected the T3 and T5 papaya germplasm resources and planted them in the papaya germplasm resource nursery in Guangdong Province, China. Two-month-old tissue-cultured T3 and T5 seedlings were obtained according to our previous description [7]. All four samples were hermaphroditic (HSY). One-way analysis of variance (ANOVA) was performed using the statistical program SPSS (v25.0) to calculate the significant differences between plant height, fruit weight, and yield of different samples. All measurements are expressed as the mean ± standard error of 3 replicates.

Samples for the Zhufeng, T3, and T5 genome assemblies were collected from the leaves of one-year-old papaya trees. The genomic DNA for PacBio HiFi was extracted using a QIAGEN Genomic-tip 100/G (QIAGEN, Germany) according to the manufacturer's instructions. DNA degradation and contamination were monitored by pulsed-field gel electrophoresis. DNA concentration and purity were analysed using a Qubit® DNA Assay Kit with a Qubit® 2.0 Fluorometer (Invitrogen, USA) and a NanoDrop 2000 (Thermo, USA), respectively. Qualified DNA was randomly fragmented using a Covaris ultrasonic disrupter. After magnetic bead enrichment of the DNA, standard procedures of end repair, poly-A and adaptor addition, fragment selection, PCR, and library quality assessment, the constructed libraries were sequenced using PacBio Sequel II.

Samples for Hi-C sequencing were collected from leaves of the same Zhufeng, T3 and T5 plants that were sampled for PacBio HiFi sequencing and were processed by standard procedures. Briefly, samples were first fixed with paraformaldehyde. Then, after cell disruption, breaks made by the 4-cutter restriction enzyme MboI (400 units) were repaired, and the labelled biotin was marked at the end of the oligonucleotide. Adjacent DNA fragments were then ligated by polynucleotide ligase. Later, during DNA purification and shearing with protease, the biotin was pulled down. Finally, the genomic DNA was divided into 350-bp fragments for sequencing [45].

### Genome assembly

For the Zhufeng, T3, T5, and Zihui varieties, PacBio data were *de novo* assembled using Hifiasm (v0.19.5) [46] with default parameters. The resulting contigs were aligned against bacterial and papaya plastid genomes using BLASTN (v2.2.30+) [47], and contigs with more than 70% of their sequence showing >95% identity to bacterial or plastid genomes were discarded. Hi-C reads were applied to achieve chromosome-level assemblies. Briefly, Hi-C reads were aligned to the assembled contigs using BWA (v0.7.17) [48] with default parameters, after which Yahs (v1.2a.1) [49] was used to cluster and order the contigs into nine superscaffolds. Genome-wide Hi-C contact maps were generated using Juicer (v1.1) [50] and visualized in JuiceBox (v1.11.08) to identify and correct potential assembly errors. The raw Sunset and SunUp genomes were aligned to the Zhufeng genome using Minimap2 (v2.26) [51], followed by correction and orientation to chromosomes using the reference-guided software RagTag with default parameters to resolve potential assembly errors in Sunset and SunUp.

### Genome annotation

RNA-seq data for 27 papaya samples across four different tissues (fruit, root, sap, and leaf) were obtained from NCBI (BioProject: PRJNA470602). Transcriptome assembly was performed using Trinity (v2.1.1) [52] and SOAPdenovo (v1.03) [53], with the results from both software programs merged and then deduplicated using cd-hit (v4.8.1) [54]. To identify transposable elements (TEs), known repeat sequences were annotated using an *ab initio* repeat library generated by RepeatMasker (v4.1.2) [55] and RepeatModeler (v1.0.8) [56], whereas tandem repeats were identified using Tandem Repeats Finder (v4.09) [57]. Before annotation, all repeats were masked on the genome. Protein-coding genes for each assembly were predicted using MAKER2 (v3.01.03) [58], retaining only sequences longer than 50 amino acids with an AED value of less than 0.5. GO terms for each gene were obtained using eggnog-mapper (v2.1.12) [59], whereas KEGG pathway annotation was performed using KOBAS (v3.0) [60] to identify potential gene functions. Protein sequences were locally aligned to the Pfam-A.hmm file downloaded from the Pfam database [61] using HMMER (v3.3.2) [62] to identify the domains of each gene.

### Syntelog-based pangenome construction

To identify core, dispensable, and private genes, we constructed a syntelog-based pangenome using the homology-based gene family clustering tool SynPan [43]. Briefly, homologous genes were first identified using Diamond (v2.1.10.164) [63] and DAGchainer (r02-06-2008) [64]. SynPan was then used to iteratively merge homologous gene pairs into a pangenome, starting with the Zhufeng genome as the initial framework. If a gene from an additional genome was homologous to any previously merged genes in the pangenome, it was assigned to an existing SG. If a gene from an additional genome was not homologous to any genes in the merged iterative pangenome, a new SG was created. SGs present in all six papaya genomes (Zhufeng, T3, T5, Zihui, Sunset, and SunUp) were defined as core SGs, SGs unique to a single papaya genome were defined as private SGs, and the remaining SGs were classified as dispensable SGs. The genes in core SGs, dispensable SGs and private SGs were defined as core genes, dispensable genes and private genes, respectively.

GO, KEGG, and PFAM enrichment analyses were performed for genes in the core, dispensable, and private SGs using the R package clusterProfiler [65]. The Ka/Ks ratios of the core and dispensable genes were calculated using KAKS_CALCULATOR [66] to estimate the selective pressures acting on these SGs.

### Graph-based pangenome construction and SV genotyping

Six papaya genomes were integrated into a multi-assembled graph using minigraph (v0.20) [67] with the following parameters: -inv no -xggs -L 10. The reference genome, Zhufeng, served as the backbone of the graph, with the remaining five genomes added sequentially. SV information within the multi-assembled graph was extracted using the bubble popping algorithm from gfatools (v0.5) [67]. Each bubble represented an SV, containing the start and end nodes of the reference sequence and the paths traversing these nodes. If two paths were observed within a bubble, the SV was classified as biallelic; if more than two paths were present, it was classified as multiallelic.

We performed SV genotyping on 222 resequenced papaya accessions and the six genomes used for graph construction using PanPop [68] to obtain high-confidence SVs. These data are divided into four populations (Common, Solo, Near-Wild, Wild) based on our previous research. Near-Wild represents a unique type of germplasm that can neither be clustered with Solo nor with Wild [7]. The population-scale SVs were then subjected to PCA and population structure analysis using GCTA (v1.94.1) [69] and ADMIXTURE (v1.3.0) [70], respectively. A neighbour-joining tree was constructed using PHYLIP (v3.696) [71] with 100 bootstraps, and the tree layout was visualized using the online tool iTOL (https://itol.embl.de/upload.cgi). The fixation index (Fst) for each SV was calculated using VCFtools (v0.1.16) [72].

### Linear pangenome construction and SV genotyping

The linear pangenome was constructed using the iterative software PSVCP (v1.01) [33]. Briefly, the reference genome Zhufeng was designated as ref0. Pairwise collinearity comparisons were performed between each of the genomes and Zhufeng using MUMmer (v4.0.0) [73] with the following parameters: -maxgap 500, -mincluster 1000, -diagdiff 20. Variants detected by MUMmer were analysed using Assemblytics (v1.1.2) [74]. SVs were identified by comparing the first genome to the Zhufeng reference genome (ref0). Insertions larger than 50 bp were integrated to create a new reference genome (ref1). This process was iteratively repeated by comparing ref1 to the next genome until all the genomes were incorporated into the pangenome. Using the constructed pangenome as the reference, SV genotyping was performed on 222 resequenced accessions using delly (v1.2.6) [75].

### Microscopy and phenotypic analyses

Preparation and photography of root paraffin sections: Forty roots each from T3 and Zhufeng seedlings were collected, embedded in paraffin, and subsequently sectioned. These sections were then stained with toluidine blue, observed, and photographed under a light microscope. Thirty sections with intact root cell structures and uniform staining were selected from each variety for subsequent analysis of cell number and size.

Phenotypic analyses: seedlings were imaged with an EOS 7D Mark II digital camera (Canon). Sixty seedlings each of T3 and Zhufeng papaya, grown under same conditions with uniform growth potential, were selected for root length statistics and analysis. The number of cells in the root meristem was defined as the quantity of cells in the middlemost layer within a 0.2-mm region of the same area in both T3 and Zhufeng. The cell length was calculated as the average length of all cells in this layer, which was measured using ImageJ software. The statistical significance of mean differences (*P* < 0.01) was analysed by Student's *t*-test.

### Polymerase chain reaction (PCR) verification and sequencing

To verify the results of the key functional gene variations revealed by the pangenome, we amplified the DNA sequences of the *BGAL15* (*Cpa15069*) from T3 and the *BGAL7* (*Cpa15078*) from Zhufeng. We also amplified the DNA of the *RRG* gene from Zhufeng, Zihui, T3, and T5 papaya varieties to determine the 94-bp sequence differences in the intron regions among these varieties. Simultaneously, we amplified the CDS sequences of the *RRG* from these same varieties to clarify whether the 94-bp sequence differences in the intron affected their CDS sequences. PCR amplifications were performed using KOD FX polymerase (TOYOBO) with specific primers. After detection by agarose gel electrophoresis and purification, the PCR amplification products were sent to Sangon Biotech Co., Ltd. for Sanger sequencing to accurately determine the gene sequences. The primer sequences are listed in Table S12.

### Quantitative real-time PCR (RT-qPCR) analysis

Total RNA was extracted from various organs using an RNeasy Plant Mini Kit (Qiagen) and reverse transcribed into cDNA using HiScript II Q Select RT SuperMix (Vazyme Biotech). RT-qPCR was carried out according to our previous description [44]. The relative gene expression level was calculated by the 2^−ΔΔCt^ method. Eukaryotic initiation factor 4A (*EIF4A*) was used as an internal control. Primer Premier 5.0 was used to design primers for RT-qPCR. The primers used were synthesized by Sangon Biotech (Table S12).

## Supplementary Material

Web_Material_uhaf282

## Data Availability

All sequencing data generated in this study were deposited at the National Center for Biotechnology Information (NCBl) under BioProject ID PRJNA1154410. The sequencing data of Zihui were downloaded from the NCBI (BioProject: PRJNA968045). The whole-genome resequencing was downloaded from the National Centre for Biotechnology Information (NCBI) under BioProject ID PRJNA970517.

## References

[1] Zhou Z, Ford R, Bar I. et al. Papaya (*Carica papaya* L.) flavour profiling. Genes (Basel). 2021;12:1416 34573398 10.3390/genes12091416 PMC8471406

[2] Aravind G, Bhowmik D, Duraivel S. et al. Traditional and medicinal uses of *Carica papaya*. J Med Plants Stud. 2013;1:7–15

[3] Yang M. et al. Comparative transcriptomics and genomic analyses reveal differential gene expression related to *Colletotrichum brevisporum* resistance in papaya (*Carica papaya* L.). Front Plant Sci. 2022;13:1038598 36618670 10.3389/fpls.2022.1038598 PMC9816866

[4] Ming R, Hou S, Feng Y. et al. The draft genome of the transgenic tropical fruit tree papaya (*Carica papaya* Linnaeus). Nature. 2008;452:991–6 18432245 10.1038/nature06856 PMC2836516

[5] Yue J, VanBuren R, Liu J. et al. SunUp and Sunset genomes revealed impact of particle bombardment mediated transformation and domestication history in papaya. Nat Genet. 2022;54:715–24 35551309 10.1038/s41588-022-01068-1

[6] Liao Z, Zhang X, Zhang S. et al. Structural variations in papaya genomes. BMC Genomics. 2021;22:335 33971825 10.1186/s12864-021-07665-4 PMC8108470

[7] Yang M, Kong X, Zhou C. et al. Genomic insights into the domestication and genetic basis of yield in papaya. Hortic Res. 2025;12:uhaf045 40236729 10.1093/hr/uhaf045 PMC11997427

[8] Huang L, Tao S, Pan Y. et al. Molecular mechanisms of low temperature-induced aberrant chilling injury in papaya fruit: physiological and transcriptomic analysis on cell wall metabolism. Sci Hortic (Amsterdam). 2025;344:114107

[9] Braga CS, Ramos HCC, Santos JS. et al. Effect of papaya ringspot virus infection in Brazilian *Carica papaya* accessions under controlled conditions. Genet Resour Crop Evol. 2025;72:7223–33

[10] Liu Y, du H, Li P. et al. Pan-genome of wild and cultivated soybeans. Cell. 2020;182:162–176.e13 32553274 10.1016/j.cell.2020.05.023

[11] Xu K, Xu X, Fukao T. et al. Sub1A is an ethylene-response-factor-like gene that confers submergence tolerance to rice. Nature. 2006;442:705–8 16900200 10.1038/nature04920

[12] Cook DE, Lee TG, Guo X. et al. Copy number variation of multiple genes at rhg1 mediates nematode resistance in soybean. Science. 2012;338:1206–9 23065905 10.1126/science.1228746

[13] Hufford MB, Xu X, van Heerwaarden J. et al. Comparative population genomics of maize domestication and improvement. Nat Genet. 2012;44:808–11 22660546 10.1038/ng.2309 PMC5531767

[14] Deng Y, Zhai K, Xie Z. et al. Epigenetic regulation of antagonistic receptors confers rice blast resistance with yield balance. Science. 2017;355:962–5 28154240 10.1126/science.aai8898

[15] Lye ZN, Purugganan MD. Copy number variation in domestication. Trends Plant Sci. 2019;24:352–65 30745056 10.1016/j.tplants.2019.01.003

[16] Li D, Wang Y, Yuan T. et al. Pangenome and genome variation analyses of pigs unveil genomic facets for their adaptation and agronomic characteristics. iMeta. 2024;3:e257 39742300 10.1002/imt2.257 PMC11683468

[17] Zhang C, Shao Z, Kong Y. et al. High-quality genome of a modern soybean cultivar and resequencing of 547 accessions provide insights into the role of structural variation. Nat Genet. 2024;56:2247–58 39251789 10.1038/s41588-024-01901-9

[18] Yang L, He W, Zhu Y. et al. GWAS meta-analysis using a graph-based pan-genome enhanced gene mining efficiency for agronomic traits in rice. Nat Commun. 2025;16:3171 40180959 10.1038/s41467-025-58081-1 PMC11968974

[19] Li P, Quan X, Jia G. et al. RGAugury: a pipeline for genome-wide prediction of resistance gene analogs (RGAs) in plants. BMC Genomics. 2016;17:852 27806688 10.1186/s12864-016-3197-x PMC5093994

[20] Nasir A, Kim KM, Caetano-Anollés G. Global patterns of protein domain gain and loss in superkingdoms. PLoS Comput Biol. 2014;10:e1003452 24499935 10.1371/journal.pcbi.1003452 PMC3907288

[21] De La Peña R, Hodgson H, Liu JC-T. et al. Complex scaffold remodeling in plant triterpene biosynthesis. Science. 2023;379:361–8 36701471 10.1126/science.adf1017 PMC9976607

[22] Huang , Jiang T, Liu Y-X. et al. A specialized metabolic network selectively modulates Arabidopsis root microbiota. Science. 2019;364:eaau6389 31073042 10.1126/science.aau6389

[23] Si L, Meng K, Tian Z. et al. Triterpenoids manipulate a broad range of virus-host fusion via wrapping the HR2 domain prevalent in viral envelopes. Sci Adv. 2024;4:eaau8408 10.1126/sciadv.aau8408 PMC6248931 30474060

[24] Srivastava G, Vyas P, Kumar A. et al. Unraveling the role of cytochrome P450 enzymes in oleanane triterpenoid biosynthesis in arjuna tree. Plant J. 2024;119:2687–705 39072959 10.1111/tpj.16942

[25] Ahn YO, Zheng M, Bevan DR. et al. Functional genomic analysis of Arabidopsis thaliana glycoside hydrolase family 35. Phytochemistry. 2007;68:1510–20 17466346 10.1016/j.phytochem.2007.03.021

[26] Liang X, Duan Q, Li B. et al. Genomic structural variation contributes to evolved changes in gene expression in high-altitude Tibetan sheep. Proc Natl Acad Sci. 2024;121:e2322291121 38913905 10.1073/pnas.2322291121 PMC11228492

[27] Pagani F, Buratti E, Stuani C. et al. A new type of mutation causes a splicing defect in ATM. Nat Genet. 2002;30:426–9 11889466 10.1038/ng858

[28] Zhou X, Li Q, Chen X. et al. The Arabidopsis retarded root growth gene encodes a mitochondria-localized protein that is required for cell division in the root meristem. Plant Physiol. 2011;157:1793–804 21984726 10.1104/pp.111.185827 PMC3327206

[29] Schuhmann H, Huesgen PF, Gietl C. et al. The DEG15 serine protease cleaves peroxisomal targeting signal 2-containing proteins in Arabidopsis. Plant Physiol. 2008;148:1847–56 18952862 10.1104/pp.108.125377 PMC2593680

[30] Li J, Duan Y, Han Z. et al. Genome-wide identification and expression analysis of the NRAMP family genes in tea plant (*Camellia sinensis*). Plants. 2021;10:10 10.3390/plants10061055 PMC8228228 34070434

[31] Boudart G, Jamet E, Rossignol M. et al. Cell wall proteins in apoplastic fluids of *Arabidopsis thaliana* rosettes: identification by mass spectrometry and bioinformatics. Proteomics. 2005;5:212–21 15593128 10.1002/pmic.200400882

[32] Consortium EUC 3 AGS, Research TI for G, Institute KDNAR . Sequence and analysis of chromosome 3 of the plant *Arabidopsis thaliana*. Nature. 2000;408:820–3 11130713 10.1038/35048706

[33] Wang J, Yang W, Zhang S. et al. A pangenome analysis pipeline provides insights into functional gene identification in rice. Genome Biol. 2023;24:19 36703158 10.1186/s13059-023-02861-9 PMC9878884

[34] Hübner S . Are we there yet? Driving the road to evolutionary graph-pangenomics. Curr Opin Plant Biol. 2022;66:102195 35217472 10.1016/j.pbi.2022.102195

[35] Yuan Z, Luo D, Li G. et al. Characterization of the AE7 gene in Arabidopsis suggests that normal cell proliferation is essential for leaf polarity establishment. Plant J. 2010;64:331–42 21070412 10.1111/j.1365-313X.2010.04326.x

[36] Yuan L, Yang X, Ellis JL. et al. The Arabidopsis SYN3 cohesin protein is important for early meiotic events. Plant J. 2012;71:147–60 22381039 10.1111/j.1365-313X.2012.04979.x

[37] Waheed A, Rehman S, Parveen B. et al. Assessment of genetic diversity and phylogenetic relationship among brinjal genotypes based on chloroplast rps 11 gene. Genet Resour Crop Evol. 2024;71:385–95

[38] Zhou Y, Zhang Z, Bao Z. et al. Graph pangenome captures missing heritability and empowers tomato breeding. Nature. 2022;606:527–34 35676474 10.1038/s41586-022-04808-9 PMC9200638

[39] Zhang X, Chen Y, Wang L. et al. Pangenome of water caltrop reveals structural variations and asymmetric subgenome divergence after allopolyploidization. Hortic Res. 2023;10:uhad203 38046854 10.1093/hr/uhad203 PMC10689057

[40] Wang M, Li J, Qi Z. et al. Genomic innovation and regulatory rewiring during evolution of the cotton genus Gossypium. Nat Genet. 2022;54:1959–71 36474047 10.1038/s41588-022-01237-2

[41] Wang S, Qian Y-Q, Zhao R-P. et al. Graph-based pan-genomes: increased opportunities in plant genomics. J Exp Bot. 2023;74:24–39 36255144 10.1093/jxb/erac412

[42] Qin P, Lu H, du H. et al. Pan-genome analysis of 33 genetically diverse rice accessions reveals hidden genomic variations. Cell. 2021;184:3542–3558.e16 34051138 10.1016/j.cell.2021.04.046

[43] Wu D, Xie L, Sun Y. et al. A syntelog-based pan-genome provides insights into rice domestication and de-domestication. Genome Biol. 2023;24:179 37537691 10.1186/s13059-023-03017-5 PMC10401782

[44] Yang M, Zhou C, Kuang R. et al. Transcription factor CpWRKY50 enhances anthracnose resistance by promoting jasmonic acid signaling in papaya. Plant Physiol. 2024;196:2856–70 39250752 10.1093/plphys/kiae479

[45] Belaghzal H, Dekker J, Gibcus JH. Hi-C 2.0: An optimized Hi-C procedure for high-resolution genome-wide mapping of chromosome conformation. Methods. 2017;123:56–65 28435001 10.1016/j.ymeth.2017.04.004 PMC5522765

[46] Cheng H, Concepcion GT, Feng X. et al. Haplotype-resolved de novo assembly using phased assembly graphs with hifiasm. Nat Methods. 2021;18:170–5 33526886 10.1038/s41592-020-01056-5 PMC7961889

[47] Altschul SF, Gish W, Miller W. et al. Basic local alignment search tool. J Mol Biol. 1990;215:403–10 2231712 10.1016/S0022-2836(05)80360-2

[48] Li H . Aligning sequence reads, clone sequences and assembly contigs with BWA-MEM. *arXiv*. 2013;1–3

[49] Zhou C, McCarthy SA, Durbin R. YaHS: yet another Hi-C scaffolding tool. Bioinformatics. 2023;39:btac808 36525368 10.1093/bioinformatics/btac808 PMC9848053

[50] Durand NC, Robinson JT, Shamim MS. et al. Juicebox provides a visualization system for Hi-C contact maps with unlimited Zoom. Cell Syst. 2016;3:99–101 27467250 10.1016/j.cels.2015.07.012 PMC5596920

[51] Li H . Minimap and miniasm: fast mapping and de novo assembly for noisy long sequences. Bioinformatics. 2016;32:2103–10 27153593 10.1093/bioinformatics/btw152 PMC4937194

[52] Haas BJ, Papanicolaou A, Yassour M. et al. De novo transcript sequence reconstruction from RNA-seq using the Trinity platform for reference generation and analysis. Nat Protoc. 2013;8:1494–512 23845962 10.1038/nprot.2013.084 PMC3875132

[53] Xie Y, Wu G, Tang J. et al. SOAPdenovo-Trans: de novo transcriptome assembly with short RNA-Seq reads. Bioinformatics. 2014;30:1660–6 24532719 10.1093/bioinformatics/btu077

[54] Fu L, Niu B, Zhu Z. et al. CD-HIT: accelerated for clustering the next-generation sequencing data. Bioinformatics. 2012;28:3150–2 23060610 10.1093/bioinformatics/bts565 PMC3516142

[55] Tarailo-Graovac M, Chen N. Using RepeatMasker to identify repetitive elements in genomic sequences. Curr Protoc Bioinform. 2009;25:4.10.1–4.10.14 10.1002/0471250953.bi0410s25 19274634

[56] Price AL, Jones NC, Pevzner PA. De novo identification of repeat families in large genomes. Bioinformatics. 2005;21:i351–8 15961478 10.1093/bioinformatics/bti1018

[57] Benson G . Tandem repeats finder: a program to analyze DNA sequences. Nucleic Acids Res. 1999;27:573–80 9862982 10.1093/nar/27.2.573 PMC148217

[58] Holt C, Yandell M. MAKER2: An annotation pipeline and genome-database management tool for second-generation genome projects. BMC Bioinformatics. 2011;12:1–14 22192575 10.1186/1471-2105-12-491 PMC3280279

[59] Cantalapiedra CP, Hernández-Plaza A, Letunic I. et al. eggNOG-mapper v2: functional annotation, orthology assignments, and domain prediction at the metagenomic scale. Mol Biol Evol. 2021;38:5825–9 34597405 10.1093/molbev/msab293 PMC8662613

[60] Xie C, Mao X, Huang J. et al. KOBAS 2.0: a web server for annotation and identification of enriched pathways and diseases. Nucleic Acids Res. 2011;39:W316–22 21715386 10.1093/nar/gkr483 PMC3125809

[61] Finn RD, Coggill P, Eberhardt RY. et al. The Pfam protein families database: towards a more sustainable future. Nucleic Acids Res. 2016;44:D279–85 26673716 10.1093/nar/gkv1344 PMC4702930

[62] Finn RD, Clements J, Eddy SR. HMMER web server: interactive sequence similarity searching. Nucleic Acids Res. 2011;39:W29–37 21593126 10.1093/nar/gkr367 PMC3125773

[63] Buchfink B, Reuter K, Drost H-G. Sensitive protein alignments at tree-of-life scale using DIAMOND. Nat Methods. 2021;18:366–8 33828273 10.1038/s41592-021-01101-x PMC8026399

[64] Haas BJ, Delcher AL, Wortman JR. et al. DAGchainer: a tool for mining segmental genome duplications and synteny. Bioinformatics. 2004;20:3643–6 15247098 10.1093/bioinformatics/bth397

[65] Yu G, Wang L-G, Han Y. et al. clusterProfiler: an R Package for comparing biological themes among gene clusters. Omi A J Integr Biol. 2012;16:284–7 10.1089/omi.2011.0118 PMC3339379 22455463

[66] Wang D, Zhang Y, Zhang Z. et al. KaKs_Calculator 2.0: a toolkit incorporating gamma-series methods and sliding window strategies. Genom Proteom Bioinform. 2010;8:77–80 10.1016/S1672-0229(10)60008-3 PMC5054116 20451164

[67] Li H, Feng X, Chu C. The design and construction of reference pangenome graphs with minigraph. Genome Biol. 2020;21:265 33066802 10.1186/s13059-020-02168-z PMC7568353

[68] Zheng Z, Zhu M, Zhang J. et al. A sequence-aware merger of genomic structural variations at population scale. Nat Commun. 2024;15:960 38307885 10.1038/s41467-024-45244-9 PMC10837428

[69] Yang J, Lee SH, Goddard ME. et al. GCTA: a tool for genome-wide complex trait analysis. Am J Hum Genet. 2011;88:76–82 21167468 10.1016/j.ajhg.2010.11.011 PMC3014363

[70] Tang H, Peng J, Wang P. et al. Estimation of individual admixture: analytical and study design considerations. Genet Epidemiol. 2005;28:289–301 15712363 10.1002/gepi.20064

[71] Felsenstein J . PHYLIP (Phylogeny Inference Package) v. 3.6. Seattle, WA: Department of Genome Sciences, University of Washington; 2005:

[72] Danecek P, Auton A, Abecasis G. et al. The variant call format and VCFtools. Bioinformatics. 2011;27:2156–8 21653522 10.1093/bioinformatics/btr330 PMC3137218

[73] Marçais G, Delcher AL, Phillippy AM. et al. MUMmer4: a fast and versatile genome alignment system. PLoS Comput Biol. 2018;14:e1005944 29373581 10.1371/journal.pcbi.1005944 PMC5802927

[74] Nattestad M, Schatz MC. Assemblytics: a web analytics tool for the detection of variants from an assembly. Bioinformatics. 2016;32:3021–3 27318204 10.1093/bioinformatics/btw369 PMC6191160

[75] Rausch T, Zichner T, Schlattl A. et al. DELLY: structural variant discovery by integrated paired-end and split-read analysis. Bioinformatics. 2012;28:i333–9 22962449 10.1093/bioinformatics/bts378 PMC3436805

