# Supplementary figures and images for: The pangenome enhances the understanding of the genetic diversity of papaya
Source: Hortic Res. 2025 Oct 16;13(2):uhaf282. doi: 10.1093/hr/uhaf282 (PMC12893817; doi:10.1093/hr/uhaf282)

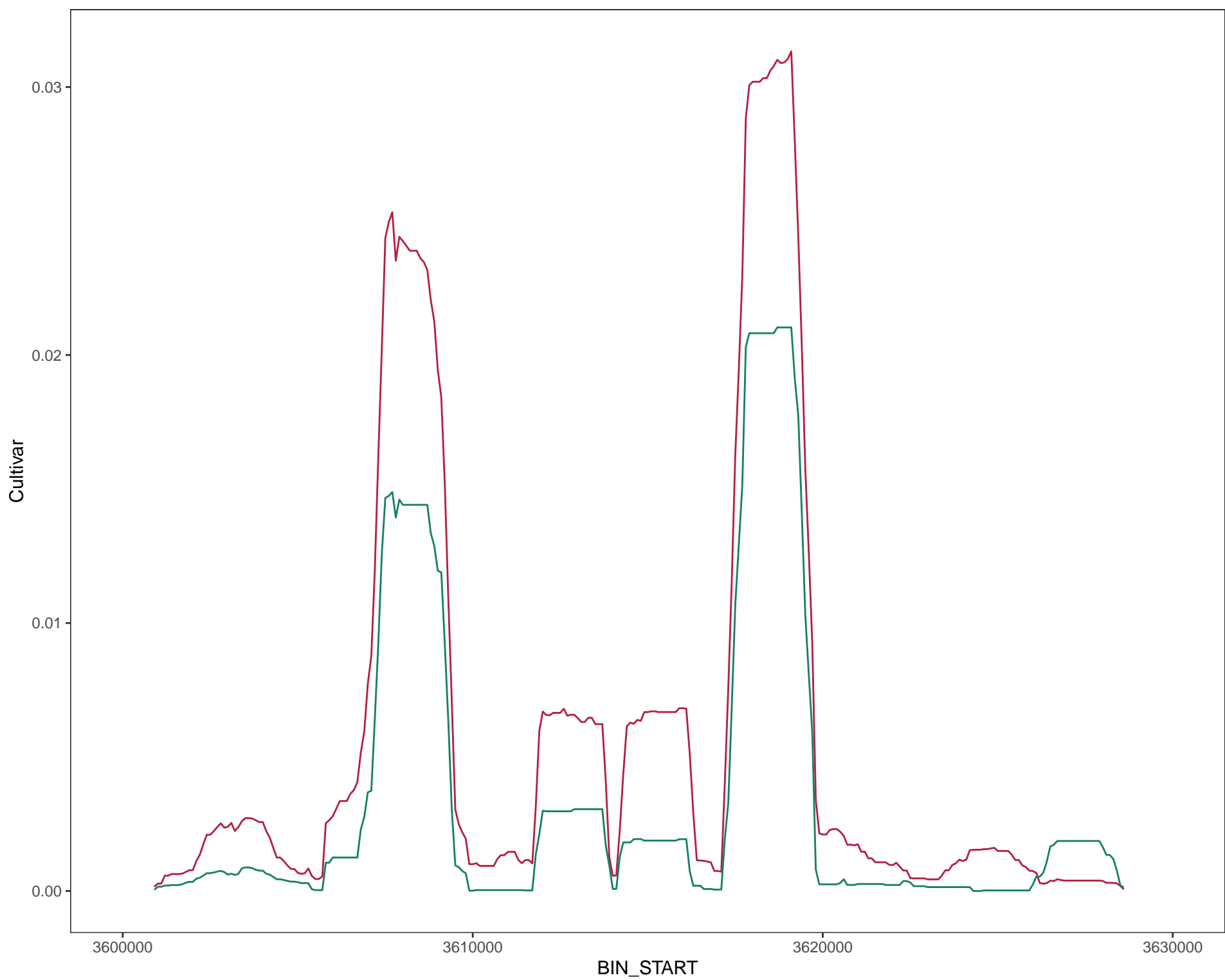

Supplement: Web_Material_uhaf282 [file web_material_uhaf282.zip › Figure S7.pdf]

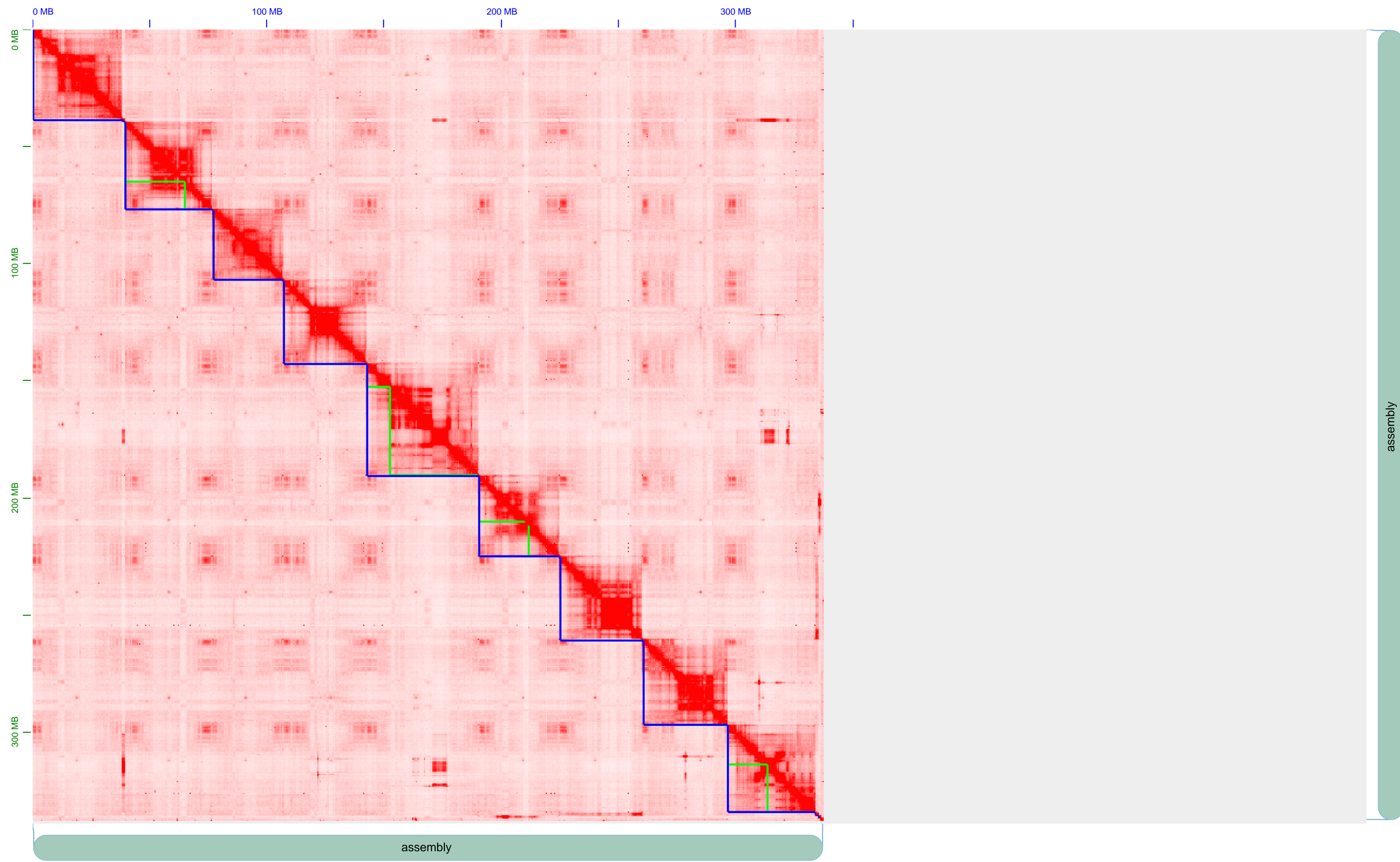

Supplement: Web_Material_uhaf282 [file web_material_uhaf282.zip › Figure S1.pdf]

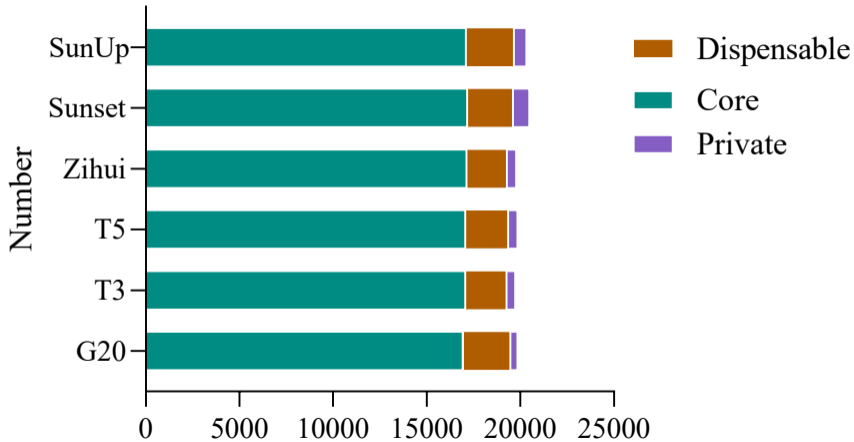

Supplement: Web_Material_uhaf282 [file web_material_uhaf282.zip › Figure S3.pdf]

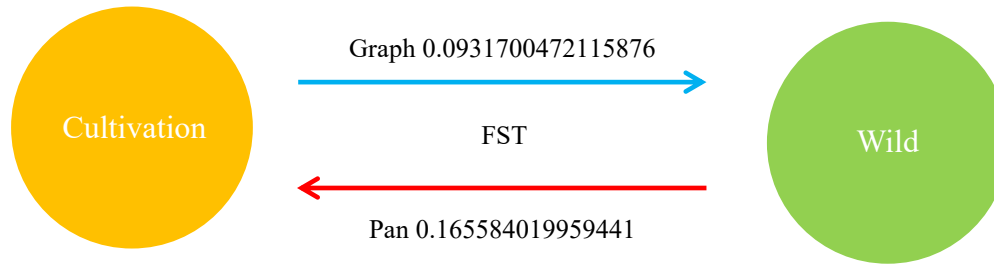

Supplement: Web_Material_uhaf282 [file web_material_uhaf282.zip › Figure S6.pdf]

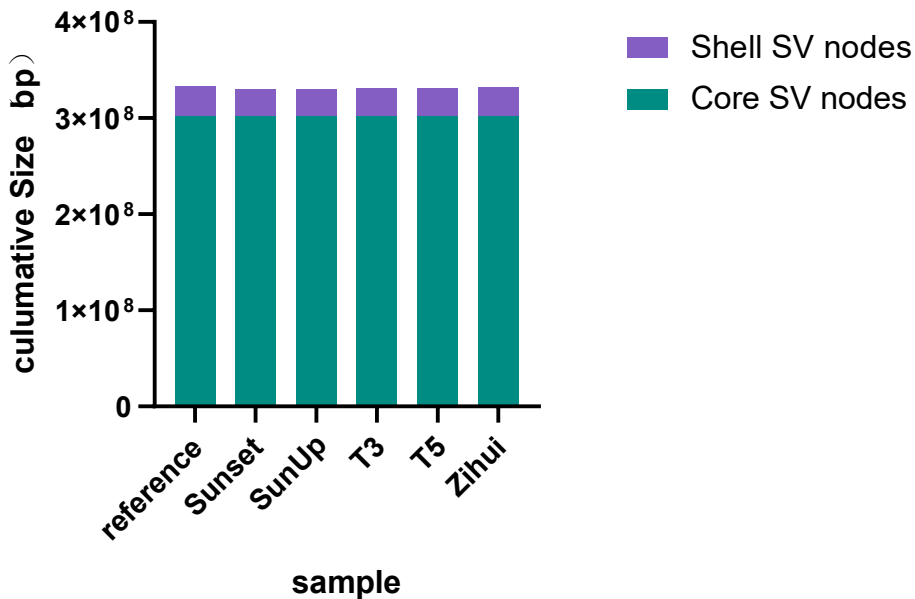

Supplement: Web_Material_uhaf282 [file web_material_uhaf282.zip › Figure S4.pdf]

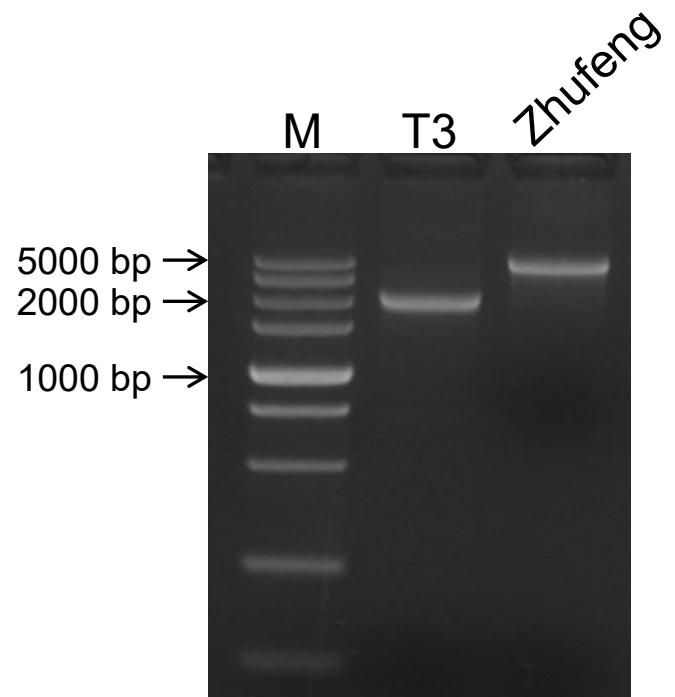

Supplement: Web_Material_uhaf282 [file web_material_uhaf282.zip › Figure S5.pdf]

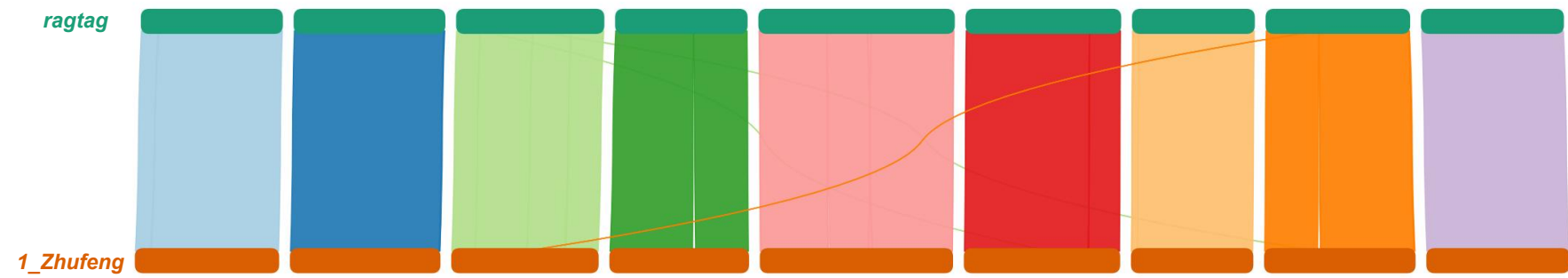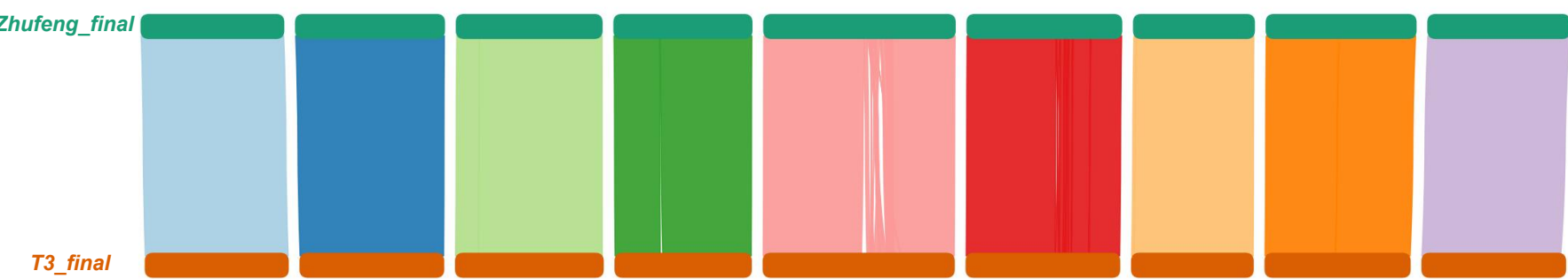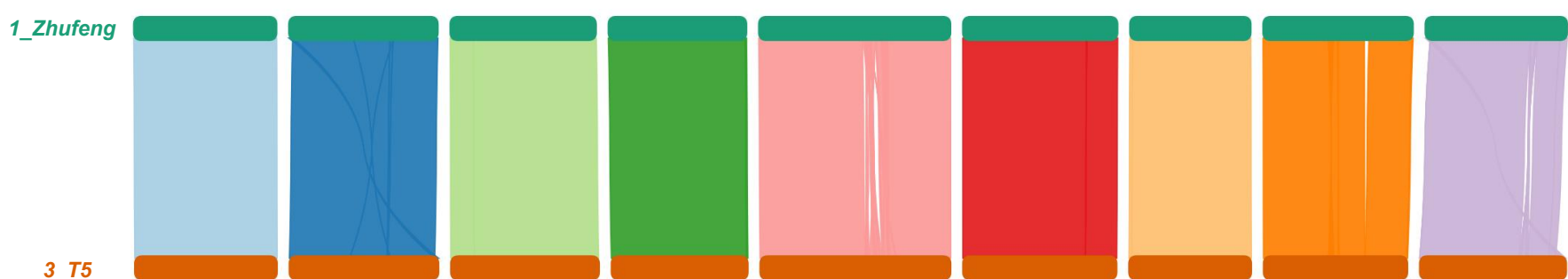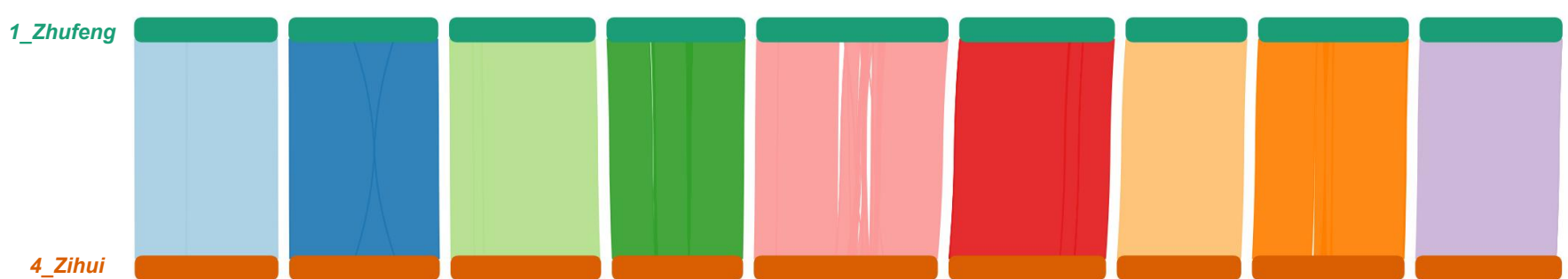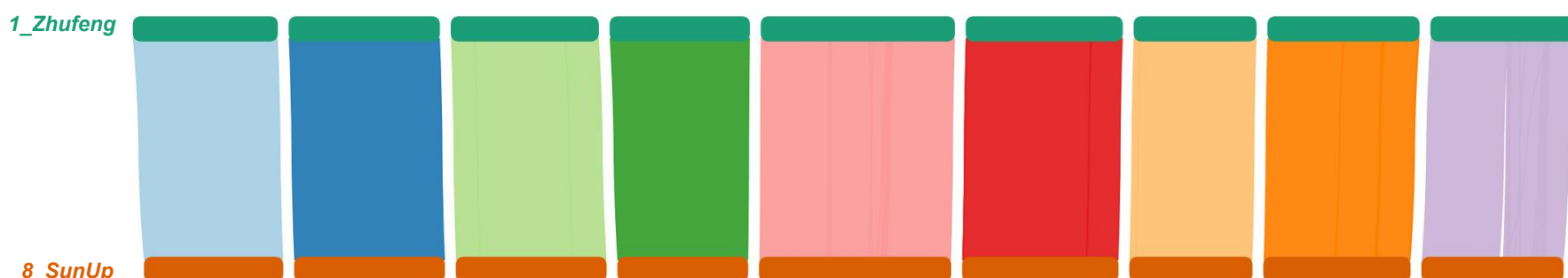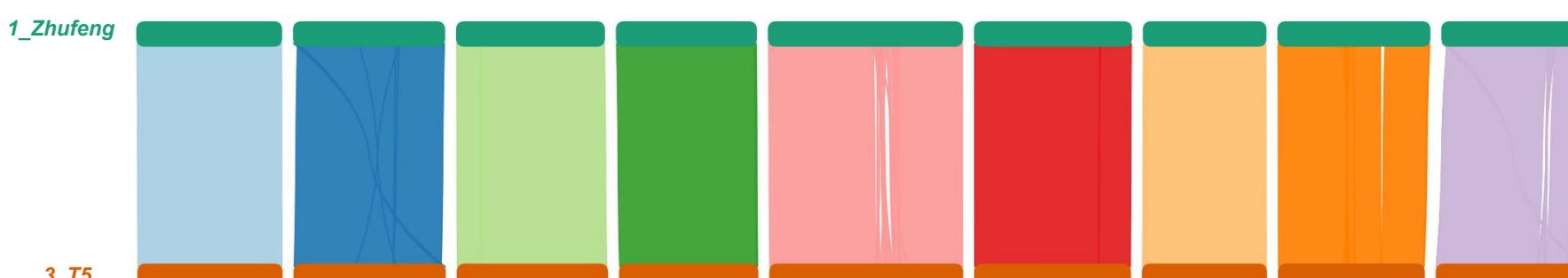

Supplement: Web_Material_uhaf282 [file web_material_uhaf282.zip › Figure S2.pdf]
